# Hemodynamic and developmental biomarkers enhance prenatal coarctation prediction: a validated multiparametric ultrasound model

**DOI:** 10.1007/s00404-025-08204-2

**Published:** 2025-10-17

**Authors:** Yu Wang, Shuhua Luo, Weiqiang Ruan, Nan Guo

**Affiliations:** 1 Department of Ultrasonic Medicine, West China Second University Hospital, Sichuan University, Chengdu, 610041 Sichuan China; 2 Key Laboratory of Birth Defects and Related Diseases of Women and Children, Sichuan University, Ministry of Education, Chengdu, 610041 Sichuan China; 3 Department of Pediatric Cardiovascular Surgery, Children’s Heart Center, West China Second University Hospital, Sichuan University, Chengdu, 610041 Sichuan China

**Keywords:** Aortic coarctation, Prenatal diagnosis, Echocardiography, Fetal growth, Hemodynamic

## Abstract

**Purpose:**

To develop and validate a prenatal prediction model for aortic coarctation (CoA) using morphologic, hemodynamic, and fetal growth parameters to enhance diagnostic accuracy and guide clinical decision-making.

**Method:**

Eighty-three fetuses with suspected CoA were retrospectively analyzed. Key prenatal predictors were analyzed using multivariable logistic regression to construct a nomogram. Model performance was evaluated via area under the receiver operating characteristic curve (AUC), calibration plots, and decision curve analysis (DCA).

**Results:**

Of the 83 fetuses, 28 (33.7%) were postnatally confirmed with CoA. The final model identified abdominal-to-head circumference ratio × 100% (*β* = 0.90, 95% CI 0.22–1.58), maximum aortic arch z-score (*β* = − 0.85, 95% CI − 1.50 to − 0.19), ventricular septal defect (OR = 1.85, 95% CI 1.02–3.53), and abnormal atrial hemodynamics (OR = 0.73, 95% CI 0.38–1.39) as significant predictors. The model achieved an AUC of 0.86 (95% CI 0.78–0.94), with calibration plots demonstrating strong agreement between predicted and observed probabilities. DCA confirmed clinical utility across a wide threshold range.

**Conclusions:**

This nomogram enhances CoA prediction by integrating structural and functional ultrasound markers. It offers strong diagnostic performance and practical value for prenatal risk stratification, potentially reducing false positives and unnecessary interventions.

**Supplementary Information:**

The online version contains supplementary material available at 10.1007/s00404-025-08204-2.

## What does this study add to the clinical work

**Table:** 

This study shows that integrating hemodynamic and developmental ultrasound biomarkers significantly enhances prenatal prediction of coarctation of the aorta. The validated multiparametric model improves diagnostic accuracy and offers practical guidance for risk-adapted surveillance and perinatal management.	

## Introduction

Coarctation of the aorta (CoA) is a congenital condition characterized by the narrowing of the aortic arch, constituting 7–8% of cardiac anomalies [1, 2]. Its pathogenesis involves neural crest cell migration defects and altered fetal hemodynamics [3–5].

Despite advances in prenatal imaging, fetal echocardiography continues to face diagnostic challenges. Open fetal shunts, including the ductus arteriosus (DA) and foramen ovale (FO), physiologically reduce left ventricular afterload and maintain antegrade aortic arch flow, thereby masking the true degree of isthmic narrowing, particularly in cases of mild to moderate CoA [6, 7].

Importantly, CoA is frequently a progressive disease [8]. The abrupt transition to postnatal circulation following spontaneous ductal closure can precipitate acute circulatory collapse [9–11]. While early identification allows for prostaglandin administration, stabilization, and timely surgical intervention at specialized centers, the prenatal detection rate remains suboptimal. Reported false-positive rates reach 38%, while false-negative rates range from 60 to 80%, resulting in either unnecessary terminations [12, 13] or delayed lifesaving treatment [14, 15].

Although anatomical markers remain diagnostic cornerstones [6], their isolated use does not capture the phenotypic heterogeneity of CoA. Emerging evidence also suggests that fetuses with congenital heart disease (CHD) demonstrate altered in utero growth trajectories compared to normal fetuses [16]. We hypothesize that a more comprehensive assessment incorporating fetal shunt dynamics and growth parameters may therefore enhance diagnostic precision. To address these limitations, we aim to develop and validate a multivariable prediction model that improves prenatal risk stratification by (1) reducing false-positive diagnoses through enhanced specificity and (2) enabling early identification of true CoA cases requiring neonatal surgery.

## Methods

### Study design and population

A retrospective cohort of fetuses with suspected aortic coarctation (CoA) was identified from a centralized echocardiography registry (Golden Disk, Chengdu, China) in one tertiary center between January 2016 and December 2023. Consecutive inclusion ensured that no eligible cases were omitted, and complete paired prenatal and postnatal echocardiographic data were available for all included fetuses, minimizing selection bias. Suspected CoA cases were defined as narrowed aortic arch with or without evidence of ventricular disproportion (defined as a tricuspid annulus diameter/mitral annulus diameter ratio > 1.6) on ultrasound assessment.[17, 18]. Exclusion criteria were as follows: (1) major structural cardiac anomalies (e.g., conotruncal anomalies); (2) multiple gestations; (3) extracardiac malformations affecting hemodynamics. All included fetuses underwent standardized, protocol-driven postnatal follow-up and diagnostic adjudication. This included serial echocardiographic assessments, review of surgical and clinical records, and final diagnostic classification based on predefined criteria.

### Echocardiographic protocol

Echocardiographic examinations were performed according to the International Society of Ultrasound in Obstetrics and Gynecology (ISUOG) guidelines using GE Voluson E10/Philips EPIQ 7C systems [19].

All fetal echocardiographic examinations were performed by senior sonographers using high-resolution ultrasound systems. If any essential cardiac structures could not be adequately visualized, real-time reacquisition was attempted during the same session. If image quality remained suboptimal, patients were scheduled for repeat scanning within 1–2 weeks. Only measurements with complete and diagnostic-quality imaging were included in the final analysis to ensure data reliability and consistency.

A triple-assessment methodology was adopted, maintaining an intraobserver coefficient of variation below 10%.To ensure measurement reliability, a second experienced fetal cardiologist independently re-measured a randomly selected subset of 20 cases (approximately 25% of the cohort), blinded to clinical outcomes. Interobserver agreement was assessed using the intraclass correlation coefficient (ICC), with all key parameters demonstrating excellent reproducibility (ICC > 0.85) [20]. Measurement fidelity was ensured by offline reanalysis using TomTec Image Arena 2.31 (TomTec Imaging, Germany).

Standardized parameters were stratified into four diagnostic categories:

- 1. Morphometrics

Arch and pulmonary artery anatomy:

Systolic measurements of the aortic annulus, ascending aorta (asA), and pulmonary annulus were obtained from the longitudinal outflow tract view. Inner diameters of transverse aortic arch (tAA) and aortic isthmus were measured from the sagittal arch view.

Cardiac chamber dimensions and right/left cardiac balance ratios:

Diameters of the mitral annulus (MVA) and tricuspid annulus (TVA), as well as heart chamber sizes were obtained from the apical four-chamber view during end-diastolic phase prior to atrioventricular valve closure. TVA/MVA, right/left atrial ratio (RA/LA), right/left ventricular ratio (RV/LV) and aortic/pulmonary annulus ratio (A/P) were calculated based on the measurements.

- 2. Hemodynamic profiling

Flow velocities and pressure gradients across atrioventricular and semilunar valves were recorded, maintaining insonation angles below 15° to ensure velocity accuracy.

Hemodynamic influences at the atrial level that can be relevant to the prenatal prediction of CoA were categorized as abnormal atrial hemodynamic status (AAH) [21, 22], including conditions as below: (1) shunt dysfunction: atrial septal aneurysm/redundant foramen ovale flap (ASA/RFOF); restrictive foramen ovale (RFO); (2) venous anomalies: persistent left superior vena cava with coronary sinus dilation (PLSVC-CS); total anomalous pulmonary venous connection (TAPVC); (3) inflow obstruction: left ventricular inflow obstruction (LVIO).

- 3. Growth biomarkers

Fetal biometry was systematically acquired, including biparietal diameter (BPD), head circumference (HC), abdominal circumference (AC), and femur length (FL). Gestational age was determined using the average ultrasound age derived from these measurements. Proportionality indices were computed to assess intrauterine growth patterns, including head circumference-to-femur length ratio (HC/FL), abdominal circumference-to-femur length ratio (AC/FL), and abdominal-to-head circumference ratio (AC/HC), which served as markers of potential asymmetrical growth or compensatory redistribution.

These growth-related parameters were interpreted in conjunction with umbilical artery Doppler indices, including the systolic/diastolic (S/D) ratio and pulsatility index (PI), to evaluate the interplay between placental perfusion and fetal growth trajectory.

All measurements were derived from a single prenatal scan per fetus, representing the highest-quality assessment prior to delivery.

- 4. Clinical variables

Key perinatal parameters encompassing fetal sex, gestational age at delivery, maternal age, delivery mode, and associated intracardiac anomalies were systematically documented for subsequent multivariate regression analysis. Definitive VSD diagnosis required postnatal echocardiographic confirmation, as prenatal ultrasound (utilizing color Doppler/2D imaging) demonstrated limited sensitivity for detecting small defects (< 2 mm) and hemodynamically insignificant shunts.

### Score normalization

All structural measurements were converted to gestational age- and body surface area-adjusted z-scores using the Boston Children’s Hospital z-score calculator (https://zscore.chboston.org/). Gestational age was based on institutional dating and verified using fetal biometry (FL, AC, BPD, HC). If the discrepancy exceeded 7 days, biometry-derived gestational age was used. Postnatal weight and height were also collected during echocardiographic follow-up to ensure consistent postnatal assessment.

### Postnatal validation

Postnatal confirmation of CoA was performed within the first 48 h after birth. Diagnostic criteria were based on the guidelines from the American Heart Association/American College of Cardiology Joint Committee. Postnatal echocardiographic evaluations were performed by pediatric cardiologists as part of routine neonatal care. While the assessors were not blinded to prenatal findings due to standard clinical handover protocols, final diagnostic classification was determined based on objective imaging criteria and/or surgical confirmation. In cases with inconclusive findings on echocardiography or clinical examination, computed tomographic angiography (CTA) was employed to support diagnostic accuracy. Comprehensive review of medical records, including surgical notes and follow-up assessments, ensured robust diagnostic validation.

Significant CoA was defined by at least one of the following postnatal criteria [1, 23–25]:

1. A noninvasive systolic blood pressure gradient > 20 mmHg between the upper and lower extremities;
2. A catheterization-derived peak-to-peak gradient > 20 mmHg across the coarctation site;
3. A Doppler echocardiography-derived mean gradient > 20 mmHg.

The potential influence of a patent DA was carefully considered, as spontaneous closure may alter aortic arch hemodynamics. To ensure diagnostic certainty, the final classification of true CoA was determined at 12 months of age, allowing for full ductal closure and assessment of persistent aortic narrowing [26].

### Longitudinal surveillance framework

A structured longitudinal follow-up protocol was implemented to monitor disease progression and support endpoint adjudication:

Phase 1: baseline echocardiography within 48 h postpartum.

Phase 2: serial follow-ups at 1, 3, 6, and 12 months of age.

### Endpoint adjudication

Confirmed CoA: progressive arch hypoplasia and persistent pressure gradient ≥ 20 mmHg.

Excluded CoA: pressure gradient < 20 mmHg with stable aortic anatomy at 12-month evaluation.

Postnatal classification enabled stratification into true-positive (TP) and false-positive (FP) cohorts for comparative analysis.

### Statistical analysis

All statistical analyses were performed using SPSS version 26 (IBM SPSS Statistics for Windows, Armonk, NY, USA) and R software. Continuous variables were summarized as mean ± standard deviation or median (interquartile range), depending on distribution normality. Between-group comparisons were conducted using the Student’s *t* test for normally distributed data or the Mann–Whitney *U* test for non-normal distributions. Categorical variables were analyzed using Fisher’s exact test. No a priori sample size calculation was conducted due to the retrospective design. A post hoc adequacy assessment was performed using the events-per-variable (EPV) approach. A two-tailed *p* value < 0.05 was considered statistically significant.

### Nomogram development and model validation

To identify independent predictors of postnatally confirmed CoA, multivariate logistic regression was performed. Results were presented as odds ratios (ORs) and 95% confidence intervals (CIs). For continuous outcomes, linear regression was used, with β coefficients and corresponding 95% CIs reported. Based on the significant predictors identified from multivariate logistic regression, a nomogram was developed to provide individualized risk estimation. Each predictor was assigned a point value proportional to its regression coefficient, and total scores were computed to generate a predicted probability of CoA.

The performance of the nomogram was evaluated based on discrimination, utilizing the area under the receiver operating characteristic curve (AUC); calibration, assessed via the Hosmer–Lemeshow goodness-of-fit test and calibration plots; and clinical utility, analyzed through decision curve analysis (DCA). The model underwent internal validation through the bootstrap resampling technique.

## Results

### Cohort characteristics

**Fig. 1:**
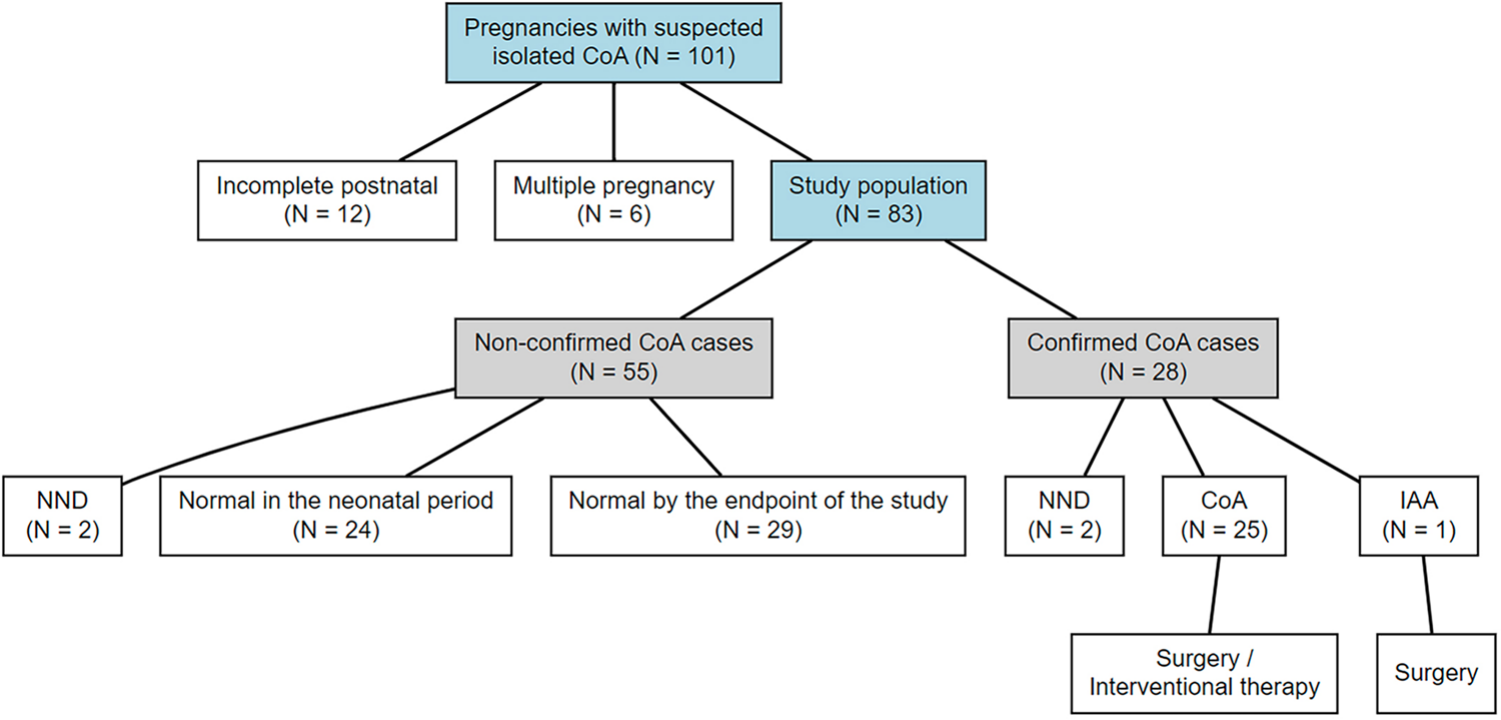
Study flowchart. Flowchart illustrating the selection and classification of pregnancies with suspected isolated coarctation of the aorta (CoA). A total of 101 fetuses with suspected CoA were screened. After exclusion of cases with multiple gestations (*n* = 6), and incomplete postnatal data (*n* = 12), 83 fetuses were consecutively included in the final cohort. These were further categorized into confirmed (*n* = 28) and non-confirmed (*n* = 55) CoA groups. Postnatal outcomes were tracked to determine final diagnoses, including neonatal death (NND), normal outcomes, and surgical interventions

A total of 101 fetuses were initially identified. After applying exclusion criteria, including 6 multiple gestations and 12 with incomplete postnatal diagnostic data, 83 fetuses with paired prenatal and postnatal echocardiographic evaluations were consecutively included in the final analysis. These cases were subsequently stratified into confirmed (*n* = 28) and non-confirmed (*n* = 55) groups (shown in Fig. 1 and Supplementary Table 1), demonstrating a PPV of 33.7% (28/83). The median gestational age at evaluation was 26.0 weeks (IQR, 24.75–29.0) in the confirmed group compared with 28.0 weeks (IQR, 26.0–30.5) in the non-confirmed group (*p* = 0.05). There were two neonatal deaths in the confirmed group (7.1%) and two in the non-confirmed group (3.6%), primarily attributed to complex congenital anomalies (total anomalous pulmonary venous connection, TAPVC in 2 cases) and respiratory failure with comorbidities. The causes of mortality were similar between groups, and the difference in mortality rates was not statistically significant (*p* = 0.612), with no deaths directly related to CoA.

### Comorbidity patterns

**Fig. 2:**
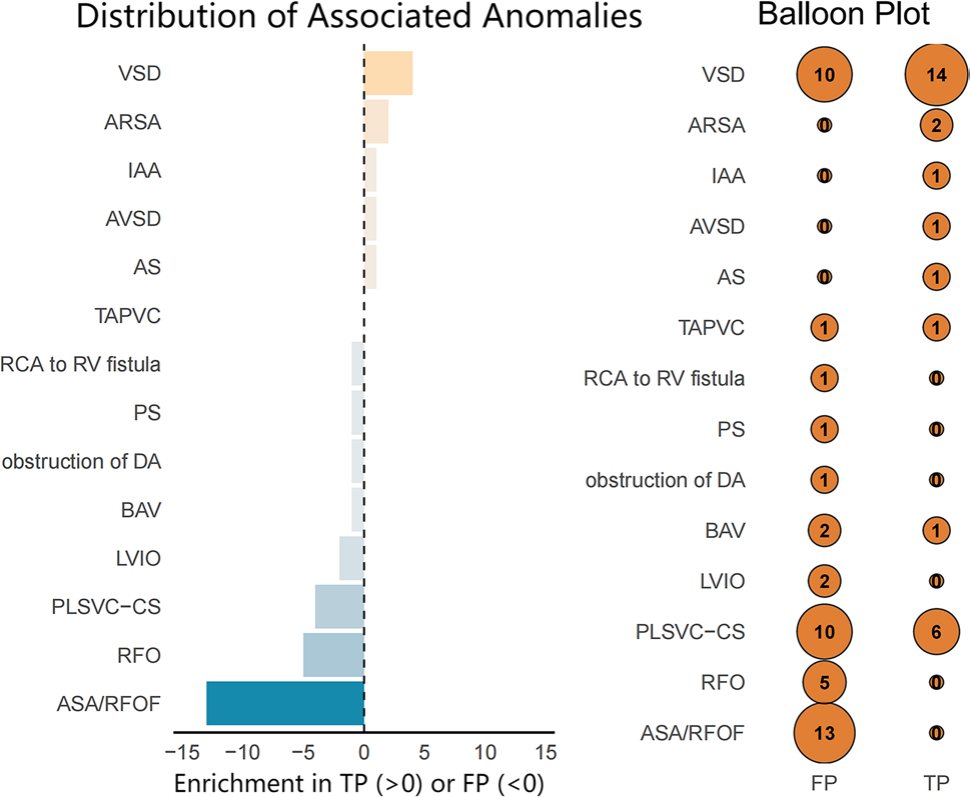
distribution of associated anomalies and balloon plot by diagnostic group. Distribution of associated anomalies and frequency of associated anomalies in confirmed (TP) and non-confirmed (FP) CoA cases. Left panel displays the difference in anomaly prevalence between groups (TP−FP), where positive values indicate associations enriched in confirmed CoA. Right panel uses balloon plot to visualize lesion frequencies. Ventricular septal defect were more frequent in confirmed cases, whereas Atrial-level hemodynamic anomaly predominated in FP diagnoses. *ARSA* aberrant right subclavian artery, *AS* aortic valve stenosis, *ASA* atrial septal aneurysm, *ASD* atrial septal defect, *AVSD* atrioventricular septal defect, *BAV* bicuspid aortic valve, *DA* ductus arteriosus, *IAA* aortic arch interruption, *LVIO* left ventricular inflow obstruction, *PLSVC-CS* persistent left superior vena cava with coronary sinus dilation, *PS* pulmonary valve stenosis, *RCA to RV* right coronary artery to right ventricle, *RFO* restrictive foramen ovale, *RFOF* redundant foramen ovale flap, *TAPVC* total anomalous pulmonary venous connection, *VSD* ventricular septal defect

Confirmed CoA cases showed unique structural anomalies: ARSA exclusively occurred in this group (7.1 vs 0%) (shown in Fig. 2 and Supplementary Table 2). VSD prevalence was higher in confirmed cases (50.0% vs 18.2%, *p* = 0.006), while AAHs predominated in non-confirmed cases (50.9 vs 25.0%, *p* = 0.034) (shown in Supplementary Table 1). Among the AAH variables, the presence of ASA/RFOF and RFO were significantly more frequent in the non-confirmed CoA group (*p* = 0.000 and *p* = 0.032, respectively; Supplementary Table 1).

### Predictive parameters

Univariate analysis identified 11 variables with significant differences between confirmed and non-confirmed CoA groups (shown in Supplementary Table 1). To minimize multicollinearity and avoid model overfitting, we selected representative variables from each diagnostic domain for inclusion in multivariate logistic regression, which included the following 4 variables (Table 1): (1) maximum aortic arch z-score (*β* = − 0.85, 95% CI − 1.58 to − 0.25, *p* = 0.01); (2) AC/HC ratio (calculated as AC/HC × 100%) (*β* = 0.91, 95% CI 0.28 to 1.67, *p* < 0.001); (3) Presence of VSD (OR = 1.85, 95% CI 1.02 to 3.53, *p* = 0.05); 4) presence of AAHs (OR = 0.73, 95% CI 0.38 to 1.39, *p* = 0.34). The final model included 4 predictors and 28 confirmed CoA cases, yielding an EPV of 7.0. This meets the widely accepted EPV guideline (≥ 5–10) for multivariate logistic regression.

**Table 1:** Multivariate logistic regression and standardized weighting of predictors for CoA

Variable	*β* value	95% CI for *β*	OR value	95%CI for OR	Standard error value	Wald value	*p* value
The max z-score	− 0.85	− 1.58 to − 0.25	0.43	0.21 to 0.78	0.34	− 2.54	0.01
AC/HC × 100%	0.91	0.28 to 1.67	2.48	1.33 to 5.32	0.35	2.60	< 0.001
Presence of AAHs	− 0.31	− 0.94 to 0.33	0.73	0.38 to 1.39	0.32	− 0.95	0.34
Presence of VSD	0.62	0.02 to 1.26	1.85	1.02 to 3.53	0.31	1.99	0.05

*AC/HC* abdominal circumference-to-head circumference ratio, *AAH* abnormal atrial hemodynamic status, *CI* confidence interval, *CoA* coarctation of the aorta, *OR* odds ratio, *VSD* ventricular septal defect

### Predictive hierarchy revealed by ROC analysis

ROC curve analysis was conducted using four selected parameters to assess their predictive value for CoA. The results are summarized in Table 2. Model 1, which included only the maximum aortic arch z-score, achieved an area under the receiver operating characteristic curve (AUC) of 0.71 (95% CI 0.59–0.82, *p* = 0.002). Model 2, which included only the AC/HC × 100% ratio, achieved an AUC of 0.76 (95% CI 0.66–0.86, *p* < 0.001). Model 3, combining the maximum aortic arch z-score and AC/HC × 100%, yielded an AUC of 0.82 (95% CI 0.73–0.91, *p* < 0.001). Model 4, the final model incorporating the maximum aortic arch z-score, AC/HC × 100%, presence of VSD, and absence of AAH, demonstrated the highest discriminative ability with an AUC of 0.86 (95% CI 0.78–0.94, *p* < 0.001).

**Table 2:** Discriminative performance of four logistic regression models for predicting postnatal coarctation of the aorta

Model	Included variables	AUC (95% CI)	*p* value
1	Maximum aortic arch z-score	0.71 (0.59–0.82)	0.002
2	AC/HC × 100%	0.76 (0.66–0.86)	< 0.001
3	Maximum aortic arch z-score + AC/HC × 100%	0.82 (0.73–0.91)	< 0.001
4	Maximum aortic arch z-score + AC/HC × 100% + presence of VSD + absence of AAH	0.86 (0.78–0.94)	< 0.001

*AC/HC* abdominal circumference-to-head circumference ratio, *VSD* ventricular septal defect, *AAH* abnormal atrial hemodynamic status, *CI* confidence interval, *AUC* area under the receiver operating characteristic curve

**Fig. 3:**
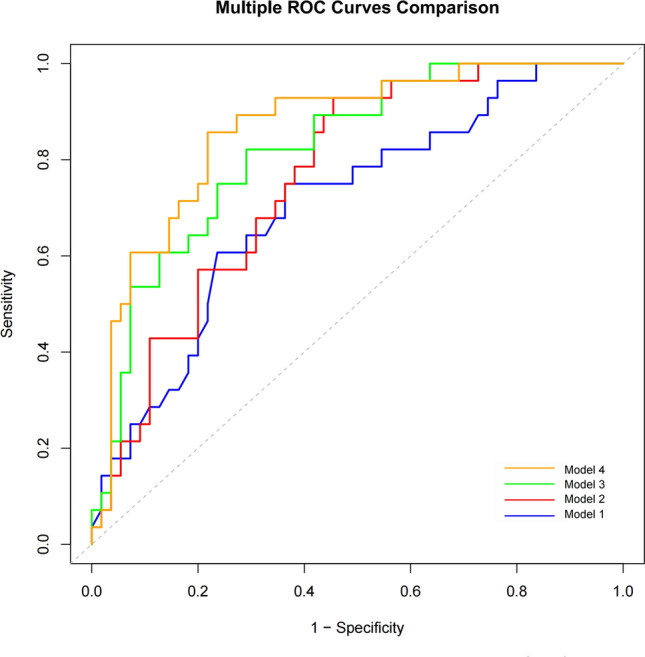
Comparative receiver operating characteristic curve analysis. Receiver operating characteristic (ROC) curves comparing the discriminative performance of four logistic regression models developed to predict postnatal coarctation of the aorta (CoA). Area under the curve (AUC) was used to quantify the diagnostic performance of each model: Model 1: Maximum aortic arch z-score only—AUC = 0.71 (95% confidence interval [CI] 0.59–0.82, *p* = 0.002). This model demonstrated the lowest predictive value with moderate diagnostic accuracy. Model 2: Abdominal circumference-to-head circumference ratio (AC/HC) × 100% only—AUC = 0.76 (95% CI 0.66–0.86, *p* < 0.001). This model provided a stronger predictive value than the maximum z-score alone. Model 3: Combination of maximum aortic arch z-score and AC/HC × 100%—AUC = 0.82 (95% CI 0.73–0.91, *p* < 0.001). This dual-parameter model further improved prediction accuracy. Model 4 (final model): Combination of maximum aortic arch z-score, AC/HC × 100%, presence of ventricular septal defect (VSD = 1), and absence of abnormal atrial hemodynamic status (AAH = 0)—AUC = 0.86 (95% CI 0.78–0.94, *p* < 0.001). This integrated model demonstrated the best discriminative ability for CoA risk stratification, highlighting the benefit of combining morphologic and hemodynamic markers

Among these, the integrated four-variable model (Model 4) demonstrated the best discriminative ability for CoA risk stratification, highlighting the benefit of combining morphologic and hemodynamic markers (Fig. 3). Although VSD and AAH were not independently significant in multivariate analysis, they were retained in the final model due to their established clinical relevance and potential mechanistic contributions to diagnostic misclassification. Their inclusion enhances the model’s clinical interpretability and ensures alignment with a mechanistically informed predictive framework.

Further, standardized logistic regression allowed quantification of each variable’s relative contribution to the model. The AC/HC × 100% contributed the most (standardized *β* = 0.91, 95% CI 0.22–1.58), accounting for 33.8% of model weight, followed by the maximum z-score (standardized *β* = − 0.85, 95% CI − 1.50 to − 0.19; 31.8%), VSD (OR = 1.85, 95% CI 1.02–3.53; 23.0%) and AAH (OR = 0.73, 95% CI 0.38–1.39; 11.5%) (Table 1).

### Model performance

**Fig. 4:**
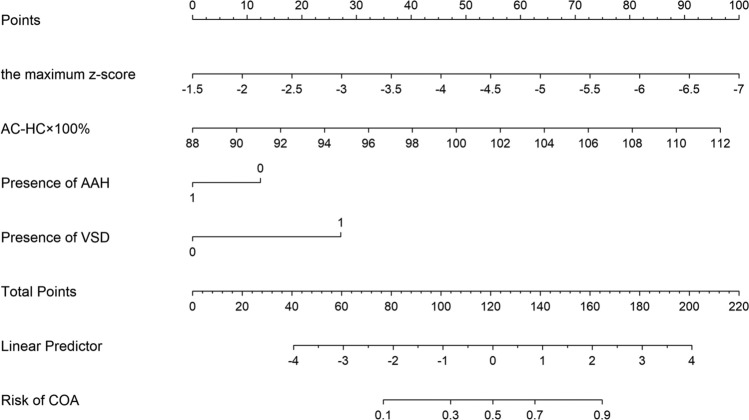
Nomogram for predicting the risk of postnatal coarctation of the aorta. Nomogram incorporating four predictors: the maximum aortic arch z-score, abdominal circumference-to-head circumference ratio (AC/HC) × 100%, presence of ventricular septal defect (VSD), and absence of abnormal atrial hemodynamic status (AAH). Each variable is assigned a score proportional to its predictive weight. The total score corresponds to a linear predictor and estimated probability of postnatal coarctation of the aorta (CoA), enabling individualized risk assessment during the prenatal period

A nomogram prediction model was subsequently developed using multivariate logistic regression (Fig. 4). At the optimal threshold determined by the maximal Youden index, the nomogram achieved a sensitivity of 85.7% and a specificity of 78.1%, indicating solid diagnostic performance for identifying fetuses at risk for CoA. The corresponding Youden index was 0.64, reflecting a favorable balance between sensitivity and specificity. This threshold was selected to maximize the true positive rate while reducing false positives, directly addressing the clinical challenge of overdiagnosis in prenatal CoA screening. At this cut-off, the model achieved a PPV of 66.7%, a negative predictive value (NPV) of 91.5%, and the number needed to test (NNT) to prevent one missed diagnosis was approximately 1.57, indicating high clinical utility. A cross-tabulation of the index test results by the results of the reference standard was constructed and shown in Table 3.

**Table 3:** Cross-tabulation of the nomogram prediction results against postnatal CoA confirmation

	CoA confirmed (*n* = 28)	Non-confirmed (*n* = 55)	Total
Test positive	24	12	36
Test negative	4	43	47
Total	28	55	83

Reference standard

*CoA* coarctation of the aorta

To further minimize overfitting and assess the model’s robustness, bootstrap resampling (1000 iterations) was conducted. The mean bootstrap AUC was 0.87 (95% CI 0.79–0.94), which was highly consistent with the original model AUC, supporting its stability and generalizability. These findings validate the model’s significant diagnostic value for CoA risk stratification in prenatal screening, supporting more targeted third-trimester surveillance and risk-adapted delivery planning for high-risk fetuses.

### Calibration and utility

**Fig. 5:**
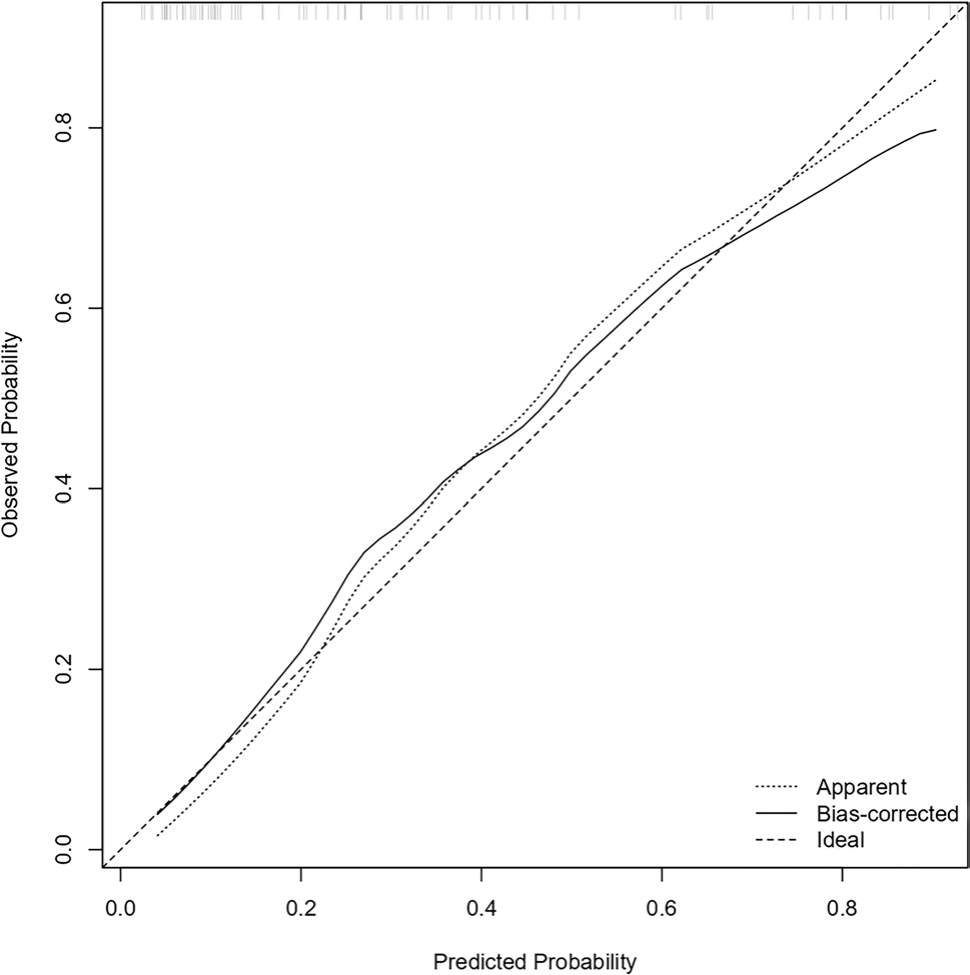
Calibration curve of the nomogram model. The calibration curve illustrates the agreement between predicted and observed probabilities of coarctation of the aorta, based on 1000 bootstrap resampling iterations for internal validation. The solid line represents bias-corrected predictions, and the dashed line represents perfect calibration. The close alignment of curves suggests good model calibration and reliable probability estimation across deciles of risk

**Fig. 6:**
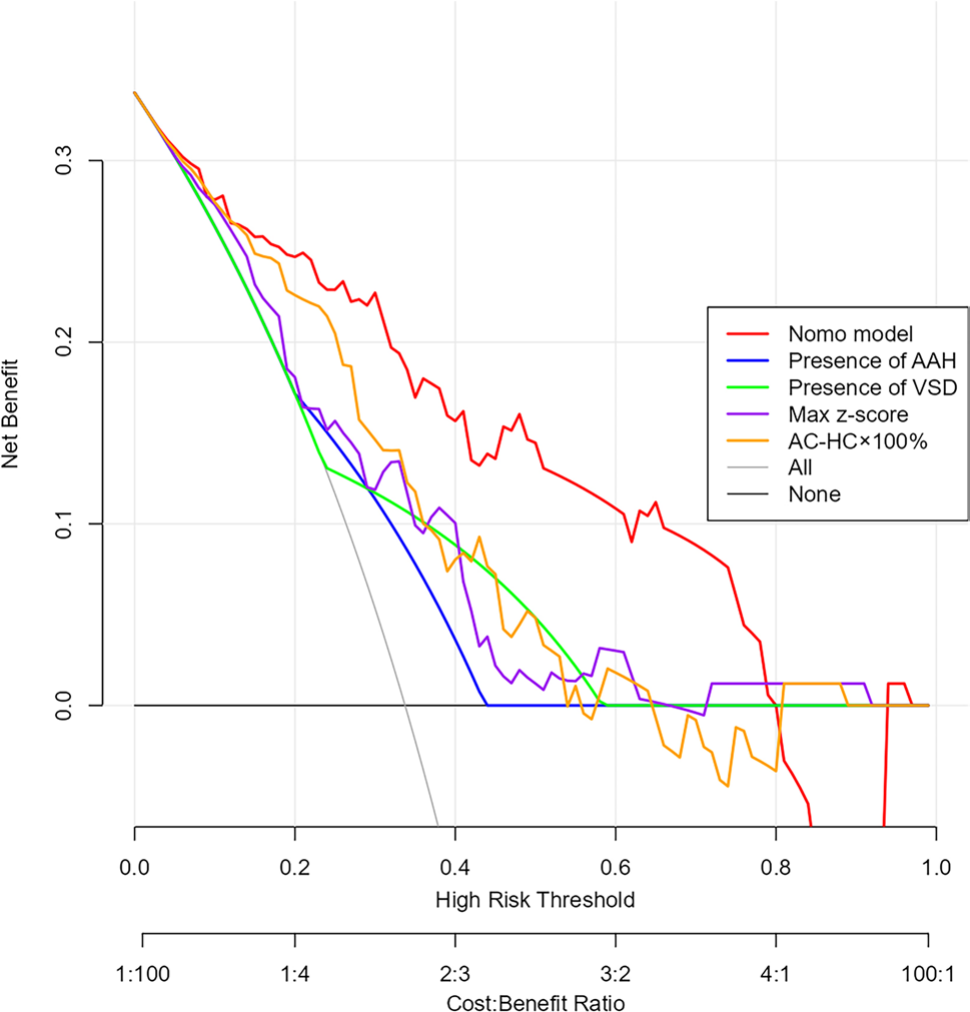
The decision curve analysis of the nomogram prediction model. The decision curve analysis (DCA) illustrates the net clinical benefit of the nomogram model (red line) compared to individual predictors, including the maximum aortic arch z-score, abdominal circumference-to-head circumference ratio (AC/HC) × 100%, presence of ventricular septal defect (VSD), and absence of abnormal atrial hemodynamic status (AAH). Reference lines for the “treat all” and “treat none” strategies are also shown. The nomogram model consistently demonstrates greater net benefit across a wide range of threshold probabilities, supporting its clinical utility in prenatal risk stratification for CoA

Hosmer–Lemeshow test (*χ*^2^ = 6.44, *p* = 0.599) and calibration curves (shown in Fig. 5) demonstrated excellent fit. Decision curve analysis (DCA), a method for evaluating the clinical net benefit of prediction models across a range of threshold probabilities, showed net benefit across thresholds (shown in Fig. 6), outperforming treat-all/no-treat strategies. The Nomo model provides significantly better clinical net benefit than a single indicator over a wide range of high-risk thresholds, supporting its use as an effective tool for CoA stratification. Prioritize the use of the Nomo model to assess fetal CoA risk, especially when the predicted risk probability is between 20 and 60% (optimal decision interval). This range reflects many borderline cases where morphologic assessment alone may be insufficient for definitive diagnosis.

## Discussion

Our study demonstrates that augmenting traditional aortic arch morphometrics (max of Z value of vessel) with hemodynamic parameters (AAH) and fetal growth indices (AC/HC × 100%) significantly improves CoA prediction. The multivariable model achieved a PPV of 66.7% versus historical morphology-only ranges of 38–60% [17, 27, 28]. This aligns with ISUOG-recommended comprehensive whole-heart phenotyping paradigm [19, 29].

The 20–60% risk interval identified by decision curve analysis offers a practical window for risk-adapted management. In this zone, the model can support clinical decisions such as referral to tertiary centers for delivery, scheduling of late-gestation follow-up scans, or closer postnatal monitoring. This is particularly valuable in settings where indiscriminate referrals may overburden high-level perinatal services, or where access to neonatal cardiac care is limited. By refining risk stratification, the model can help optimize the use of specialized resources and reduce unnecessary interventions in low-risk cases, while ensuring timely care for those at true risk.

Contemporary researches redefine CoA as a pan-aortic vasculopathy involving endothelial dysfunction and extracellular matrix dysregulation, extending beyond focal stenosis [30, 31]. While prior studies have emphasized morphological variations in arch anatomy [15, 31], our data identify maximal aortic narrowing severity as diagnostically superior to lesion location. This divergence stems from technical challenges in fetal aortic imaging: spatial overlap between the ductus arteriosus and aortic isthmus often obscures isthmic visualization, while the inherently small fetal isthmus diameter (especially in CoA fetuses < 2 mm) magnifies measurement errors [15]. Since obtaining a perfect sagittal section of the aortic arch places extremely high demands on the ultrasound operator’s technique, other alternative measurement methods have been used in the hope of obtaining more morphological information of the aortic arch, such as using the inner diameter of the junction of aorta and the ductus arteriosus on the three-vessel view to reflect the inner diameter of the isthmus or using the ratio of isthmus diameter to the diameter of the ductus arteriosus[32]. According to our experience, since the transverse aortic arch continues with the descending aorta at a certain angle and is pulled by the ductus arteriosus, the measurement of the slender transverse section of the isthmus of the aortic arch will inevitably result in large errors or mistakes. However, it is relatively easy to obtain the sagittal section of the ascending aorta and the proximal segment of the transverse arch. Therefore, in some cases of CoA combined with aortic hypoplasia, the measurement of the sagittal section of the ascending aorta and the transverse arch is more reliable. Furthermore, we have found that, unlike in the postnatal population, limited stenosis of the isthmus is comparatively rare in the fetus. We performed a univariate analysis on the z-scores of the proximal and distal aortic arch and found no statistical difference between groups (22/28 was found more narrow part is asA in TP group, *p* = 0.791). These findings highlight the importance of precise multi-segment aortic z-score calculations over traditional morphometric ratios [15], with Zmax being a marker that reflects the overall degree of stenosis and provides important information for the diagnosis of CoA, aligning with evidence of systemic aortic pathology in CoA [30].

Left–right ventricular discrepancy remains a key prenatal CoA marker, with hemodynamic imbalances driving secondary anatomical changes [10, 17, 33–35]. Our findings confirm this paradigm but reveal a subset of fetuses with atrial-level shunting anomalies (AAH-related conditions) exhibiting CoA-like asymmetry exceeding confirmed cases. This expands prior evidence of AAH-induced diagnostic uncertainty through two mechanisms: (1) restricted right-to-left shunting or (2) excessive right heart flow, both reducing aortic volume. ASA is an uncommon fetal echocardiographic finding, typically characterized by excessive excursion of the atrial septum into the LA cavity. Although generally regarded as a transient and self-limiting anomaly, it may occasionally precipitate fetal arrhythmia or obstruct LV inflow. AAH such as RFO and LVIO were frequently associated in these cases. Chronic obstruction reduces LV preload and output, initiating a pathophysiological cascade that may progress to left ventricular hypoplasia and aortic arch underdevelopment [36, 37]. Multiple case reports indicate a consistent association between isolated PLSVC-CS and CoA [38–40]. Study found that the incidence of right heart enlargement, VSD, CoA, and IAA was relatively higher in fetuses with PLSVC-CS and associated cardiac structural abnormalities [41, 42]. However, the underlying pathophysiological mechanisms driving this correlation remain incompletely elucidated. Based on postnatal LV volume recovery observed in some infants with PLSVC-CS, we hypothesize that fetal CS dilation creates a localized bulge of the FO valve toward the LA orifice. This mechanical distortion increases resistance to right-to-left shunting across the FO, thereby reducing LV preload while augmenting RV volume. Postnatal normalization occurs with the cessation of fetal circulation and closure of the FO. A parallel hemodynamic mechanism has been documented in fetuses with intracardiac anomalous pulmonary venous connection associated with CS dilation. However, the main reason for the increased right heart load of other types of TAPVC without CS dilation is the abnormal return of pulmonary blood flow to the right heart after birth. In our study, AAH-associated anomalies were significantly more prevalent in non-CoA cases (50.9 vs. 25.0%, *p* = 0.034), supporting its role in mimicking CoA via transient flow disturbances [22, 43–45]. However, AAH lost predictive significance in multivariate analysis (AUC = 0.63, 95% CI 0.51–0.75, *p* = 0.055), likely due to collinearity with aortic z-scores and dilution by stronger anatomical markers. This refines AAH’s utility as a cautionary indicator requiring corroborative aortic evidence, addressing the “overestimation paradox” from overreliance on hemodynamic markers [34, 44].

In the fetal circulation, a small, isolated VSD typically does not induce significant LV volume overload or cause ventricular disproportion. Therefore, the common presentation of RV dilation and disproportion in fetuses with CoA and concomitant small VSD suggests minimal hemodynamic impact from the VSD’s left-to-right shunt. Conversely, a large VSD may permit substantial right‐to‐left shunting at the ventricular level, potentially masking the characteristic marked right ventricular dilation and left–right cardiac disproportion typically associated with CoA. Although clinical coexistence of VSD and CoA is well-documented, the precise pathophysiological mechanisms underlying this association remain incompletely elucidated. Embryologically, it is possible that defects in neural crest cell migration may contribute to the development of both CoA and perimembranous VSD [46]. Studies examining the LV/RV ratio have reported heterogeneous findings, with significant variations in reported normal ranges and pathological thresholds across different patient populations and methodologies. Xiao et al. demonstrated ventricular disproportion’s limited standalone predictive value (AUC = 0.62) [47], while Quartermain et al. advocated combining ventricular ratios with aortic dimensions [48]. Our cohort showed higher VSD prevalence in true-positive versus false-positive CoA cases (50.0 vs. 18.2%, *p* = 0.006), which is similar to previous study (87.5 vs. 41.18%, *p* = 0.002) [49], yet neither VSD presence nor RV/LV ratio retained multivariate significance. Consistent with prior research on CoA phenotyping, VSD is increasingly recognized as a key diagnostic covariate in predictive models, reflecting its established comorbidity with aortic arch anomalies [14, 32, 50]. VSD was retained in our final model due to their established relevance in prior literature and their moderate predictive value (AUC = 0.659, 95% CI 0.529–0.789, *p* = 0.018). Including VSD enhances our model’s clinical interpretability especially in equivocal cases.

Studies have shown that fetuses with CHD have unique intrauterine growth patterns and associated with smaller HC and neurodevelopmental delay. Existing studies [16, 51, 52] have explored and verified the possible hemodynamic effects from different perspectives. The common hypothesis is that in the presence of a normal abdominal growth, HC growth is diminished in fetuses with CHD with reduced ascending aorta oxygen saturation and/or reduced aortic arch flow. While there is no special hemodynamic study on children with fetal coarctation, Sifa Turan et al. [53] reported that in left-sided CHD, lesions with reversed isthmic blood flow, such as hypoplastic left heart syndrome, are associated with delayed head growth, growth disproportion, and evidence of brain-sparing, whereas lesions with antegrade isthmic flow show brain-sparing with normal head and fetal growth. It may reflect distinct pathophysiological mechanisms underlying CoA as compared to other forms of CHD. In our study, we found a significant increase of AC/HC × 100% in confirmed CoA cases (102.61 ± 3.26) compared to non-confirmed cases (98.55 ± 4.48, *p* < 0.001). Multivariate analysis further identified AC/HC × 100% as an independent predictor of postnatal CoA (*β* = 0.90, *p* = 0.015), with moderate to strong discriminatory power in ROC analysis (AUC = 0.76, 95% CI: 0.661–0.864). This suggests that fetuses in the TP group are more likely to have delayed head growth and growth disproportion, which means that there is a high probability of varying degrees of reversed flow in the fetal aortic isthmus or descending aorta. Hiroshi Kawamura, et al.[54] conducted a retrospective study to validate the relationship between retrograde blood flow in the aortic isthmus (AoI-R) by color Doppler in fetal echocardiography and postnatal CoA diagnosed as isthmus narrowing, the result demonstrated AoI-R determined by color Doppler echocardiography can become a useful tool in the screening of fetal CoA, especially at < 35 weeks of gestation. To better understand the hemodynamic changes in fetuses with coarctation, Inmaculada Villanueva-Baxarias et al.[55] conducted a retrospective study, using a 0D computational model of fetal circulation to understand the effect of CoA-related cardiovascular remodeling, corroborated that in fetal CoA a redistribution of blood flow occurs to ensure perfusion of the brain and placenta, without a significant alteration in fetal hemodynamics (blood pressure and velocities) except for increased diastolic velocities in the AoI. These findings suggest that CoA may be characterized by a distinct and complicated fetal growth phenotype resulted in disproportionate abdominal over head growth, and highlight the importance of considering AC/HC × 100% as a potentially useful screening parameter. Future studies combining longitudinal AC/HC trajectories with advanced hemodynamic and Doppler assessments may further elucidate the mechanisms underlying this growth pattern and improve the prenatal detection of CoA.

### Strengths and limitations

This study developed a multiparametric prediction model incorporating structural, hemodynamic, and growth-related markers, with robust internal validation via bootstrap resampling. The model demonstrated good discrimination, calibration, and clinical utility within the study cohort.

However, the study was retrospective and conducted at a single tertiary center with a modest sample size. The model was developed based on 28 events (< 10 events per predictor) and underwent internal validation only; therefore, overfitting and optimism remain possible, and external validation is essential before clinical application. Additionally, variability in prenatal follow-up limited the availability of serial measurements, and some advanced imaging parameters were not routinely collected.

### Clinical implications

In the process of exploration in this study, we found that the introduction of hemodynamics and fetal growth indicators based on the traditional prenatal ultrasound study of fetal aortic arch morphology can increase the diagnostic efficacy of the disease, verifying our hypothesis; in future clinical work, we can add more commonly used and reproducible clinical indicators under these major frameworks to improve the diagnostic model of the disease. Future prospective, multicenter studies are warranted to validate and refine this prediction model.

## Conclusions

In conclusion, our study offers a comprehensive approach to prenatal CoA diagnosis, integrating echocardiographic, hemodynamic, and fetal growth parameters. By combining these markers, we improve the accuracy of CoA diagnosis and risk stratification, supporting better-targeted surveillance and perinatal management. Future research should prioritize longitudinal aortic arch assessments, dynamic growth trends, and advanced Doppler evaluations to further enhance prenatal screening accuracy and early detection.

## Supplementary Information

Below is the link to the electronic supplementary material.

Supplementary file1 (DOCX 21 KB)

Supplementary file2 (DOCX 25 KB)

## Data Availability

The datasets generated and/or analyzed during the current study are not publicly available due to the restriction by the hospital policy but are available from the corresponding author on reasonable request.
