# Supplementary material for: Hemodynamic and developmental biomarkers enhance prenatal coarctation prediction: a validated multiparametric ultrasound model
Source: Arch Gynecol Obstet. 2025 Oct 17;312(6):2175–86. doi: 10.1007/s00404-025-08204-2 (PMC12705779; doi:10.1007/s00404-025-08204-2)
Supplement: Supplementary file 1 — Supplementary file1 (DOCX 21 KB) [file 404_2025_8204_MOESM1_ESM.docx]

| Variable | Non-confirmed (n=55) | Confirmed CoA (n=28) | p-value |
| --- | --- | --- | --- |
| **Morphometrics** | | |  |
| Arch and pulmonary artery anatomy | | |  |
| More narrow part is asA | 46/55 | 22/28 | 0.791 |
| asA z-score | -2.20 ± 1.04 | -2.46 ± 1.41 | 0.400 |
| tAA z-score | -3.23 ± 1.20 | -3.83 ± 1.09 | 0.026 |
| The max z-score | -3.43 ± 0.90 | -4.13 ± 0.85 | 0.001 |
| Right/left cardiac balance ratios | | |  |
| RA/LA | 1.39 ± 0.46 | 1.47 ± 0.48 | 0.434 |
| RV/LV | 1.12 ± 0.27 | 1.20 ± 0.29 | 0.232 |
| RH/LH | 1.22 ± 0.30 | 1.29 ± 0.30 | 0.317 |
| TVA/MVA | 1.25 ± 0.28 | 1.33 ± 0.24 | 0.216 |
| A/P | -1.27 ± 0.93 | -1.74 ± 0.95 | 0.037 |
| **Hemodynamic Profiling** | | |  |
| PV (m/s) | 0.76 ± 0.14 | 0.76 ± 0.16 | 0.963 |
| AV (m/s) | 0.81 ± 0.20 | 0.80 ± 0.21 | 0.939 |
| PV/AV | 0.99 ± 0.28 | 0.98 ± 0.22 | 0.809 |
| TVe (m/s) | 0.44 ± 0.15 | 0.40 ± 0.09 | 0.097 |
| MVe (m/s) | 0.40 ± 0.11 | 0.41 ± 0.12 | 0.986 |
| TVe/MVe | 1.12 ± 0.30 | 1.01 ± 0.22 | 0.070 |
| TVa (m/s) | 0.63 ± 0.13 | 0.59 ± 0.12 | 0.144 |
| MVa (m/s) | 0.57 ± 0.14 | 0.58 ± 0.12 | 0.750 |
| TVa/MVa | 1.14 ± 0.24 | 1.02 ± 0.12 | 0.003 |
| Presence of AAHs | 28/55 | 7/28 | 0.034 |
| ASA/RFOF | 13/55 | 0/28 | 0.000 |
| RFO | 5/55 | 0/28 | 0.032 |
| PLSVC-CS | 10/55 | 6/28 | 0.782 |
| TAPVC | 1/55 | 1/28 | 1.000 |
| LVIO | 2/55 | 0/28 | 0.547 |
| **Growth Biomarkers** | | |  |
| Umbilical artery S/D | 2.40 ± 0.48 | 2.36 ± 0.33 | 0.684 |
| Placenta PI | 0.88 ± 0.20 | 0.87 ± 0.17 | 0.349 |
| HC z-score | -0.80 (-1.35–-0.10) | -0.85 (-1.60–-0.57) | 0.358 |
| FL z-score | -1.20 ± 0.67 | -1.21 ± 0.74 | 0.948 |
| AC z-score | -0.40 (-1.10–0.20) | 0.10 (-0.43–0.62) | 0.030 |
| HC/FL | 4.73 ± 0.18 | 4.64 ± 0.11 | 0.009 |
| AC/FL | 4.66 ± 0.18 | 4.77 ± 0.17 | 0.011 |
| AC/HC | 0.99 ± 0.04 | 1.03 ± 0.03 | 0.000 |
| AC/HC × 100% | 98.55 ± 4.48 | 102.61 ± 3.26 | 0.000 |
| **Clinical Variables** | | |  |
| Male | 36/55 | 13/28 | 0.153 |
| GA at examination (weeks) | 26 (28-30.5) | 24.75 (26-29) | 0.05 |
| GA of delivery (weeks) | 39(37–39) | 39(38.75–40) | 0.924 |
| Vaginal delivery | 14/55 | 14/28 | 0.053 |
| Maternal age (years) | 31(23–39) | 31(25–39) | 0.630 |
| Presence of VSD | 10/55 | 14/28 | 0.006 |
| ARSA | 0/55 | 2/28 | 0.111 |
| BAV | 2/55 | 1/28 | 1.000 |
| IAA | 0/55 | 1/28 | 0.337 |
| AVSD | 0/55 | 1/28 | 0.337 |
| obstruction of DA | 1/55 | 0/28 | 1.000 |
| PS | 1/55 | 0/28 | 1.000 |
| AS | 0/55 | 1/28 | 0.337 |
| RCA to RV fistula | 1/55 | 0/28 | 1.000 |

Supplementary Table 1 Comparison of Prenatal Echocardiographic and Clinical Parameters Between Confirmed and Non-Confirmed CoA Groups

Abbreviations:

AC, abdominal circumference; A/P, aortic/pulmonary annulus; AC/FL, abdominal circumference-to-femur length ratio; AC/HC, abdominal circumference-to-head circumference ratio; AAH, abnormal atrial

hemodynamic status; ARSA, aberrant right subclavicular artery; AS, aortic valve stenosis; asA, ascending aorta; ASA/RFOF, atrial septal aneurysm/Redundant foramen ovale flap; ASD, atrial septal defect; AV, aortic valve velocity; AVSD, atrioventricular septal defect; BAV, bicuspid aortic valve; FL, femur length; GA, gestational age; HC, head circumference; IAA, interrupted aortic arch; LA/RA, left/right atrium; LVIO, left ventricular inflow obstruction; LV/RV, left/right ventricle; MVA/TVA, mitral/tricuspid valve annulus; MVa, peak mitral a wave velocity; MVe, peak mitral e wave velocity; PI, pulsatility index; PLSVC-CS, persistent left superior vena cava with coronary sinus dilation; PS, pulmonary stenosis; PV, pulmonary valve velocity; RCA to RV, right coronary artery to right ventricle; RFO, restrictive foramen ovale; S/D, systolic/diastolic ratio; TAPVC, total anomalous pulmonary venous connection; tAA, transverse aortic arch; TVa, peak tricuspid a wave velocity; TVe, peak tricuspid e wave velocity; VSD, ventriculap septal defect.
