# Supplementary material for: Hemodynamic and developmental biomarkers enhance prenatal coarctation prediction: a validated multiparametric ultrasound model
Source: Arch Gynecol Obstet. 2025 Oct 17;312(6):2175–86. doi: 10.1007/s00404-025-08204-2 (PMC12705779; doi:10.1007/s00404-025-08204-2)
Supplement: Supplementary file 2 — Supplementary file2 (DOCX 25 KB) [file 404_2025_8204_MOESM2_ESM.docx]

Supplementary Table 2. Case-Level Summary of Prenatal Diagnosis, Postnatal Outcomes, and Associated Anomalies

| Group | Case | Gender | Prenatal diagnosis | Postnatal diagnosis | Outcome |
| --- | --- | --- | --- | --- | --- |
| Non-confirmed CoA cases | 2 | M | CoA,ASA/RFOF,RFO | PDA,PFO,SSRN. | well |
|  | 4 | M | CoA,VSD | VSD, ASD,PDA | Interventional therapy, well |
|  | 7 | F | CoA,VSD | VSD,PFO,PDA,SSRN | VSD,well |
|  | 9 | F | CoA,ASA,LVIO,RFO | ASA, ASD,SSRN,PAH | Well |
|  | 11 | M | CoA,PLSVC-CS | PLSVC-CS,PDA,ASD | Well |
|  | 12 | M | CoA, RCA to RV fistula, PS(mild) | RCA to RV fistula, PS(moderate),ASD | Interventional therapy, well |
|  | 13 | F | CoA, VSD | VSD(multiple), PDA,PFO,SSRN | Interventional therapy, well |
|  | 14 | M | CoA，VSD | VSD,SSRN | Well |
|  | 15 | M | CoA | PDA | Well |
|  | 17 | F | CoA,ASA/RFOF,RFO | PDA, PFO, SSRN. | Well |
|  | 19 | M | CoA | PFO, PDA | well |
|  | 20 | M | CoA,ASA/RFOF | TAPVC(infra cardiac),ASD,PAH | NND |
|  | 21 | M | CoA,ASA/RFOF,RFO | PDA ASD, SSRN,PAH | Interventional therapy, well |
|  | 22 | M | CoA,ASA/RFOF | PFO, SSRN | Well |
|  | 23 | M | CoA,VSD | PFO PDA VSD SSRN | well |
|  | 24 | M | CoA,mild obstruction of ductus arteriosus | PFO, PDA | well |
|  | 25 | F | CoA | PFO,PDA,SSRN | well |
|  | 29 | M | CoA,ASA/RFOF with LVIO | PDA, ASD, SSRN,PAH | NND |
|  | 30 | F | CoA,VSD, PLSVC-CS | PFO, PLSVC-CS, BAV, VSD | Well |
|  | 32 | F | CoA,ASA/ROFO | PDA | Well |
|  | 33 | M | CoA, PLSVC-CS, ASA/ROFO | PDA,PFO, PLSVC-CS, SSRN | Well |
|  | 34 | F | CoA, ASA/ROFO | PDA, PFO, ASD, SSRN | Well |
|  | 35 | F | CoA,PLSVC-CS | PLSVC-CS | Well |
|  | 37 | M | CoA | SSRN | Well |
|  | 38 | M | CoA,PLSVC-CS | PLSVC-CS | Well |
|  | 39 | F | CoA | PFO | Well |
|  | 40 | M | CoA | PDA, PFO, ASD | Well |
|  | 41 | F | CoA,PLSVC-CS | PFO, PLSVC-CS | Well |
|  | 42 | M | CoA | PFO, PDA | Well |
|  | 43 | M | CoA, ASA/RFOF | PFO, PDA, SSRN | Well |
|  | 44 | M | CoA | SSRN, BAV | Well |
|  | 49 | F | CoA,PLSVC-CS, VSD | PFO, PLSVC-CS, VSD, PDA, SSRN | VSD, Well |
|  | 50 | M | CoA, ASA/RFOF | SSRN, ASD, PDA | ASD, Well |
|  | 51 | M | CoA | SSRN, ASD, PFO, PDA | ASD, PFO, Well |
|  | 52 | F | CoA,PLSVC-CS, VSD | PFO, PLSVC-CS, VSD, PDA | Well |
|  | 56 | M | CoA | SSRN, PFO, PDA | Well |
|  | 57 | F | CoA, ASA/RFOF | SSRN, PFO, PDA | Well |
|  | 61 | M | CoA | SSRN | Well |
|  | 64 | F | CoA, PLSVC-CS | PFO, PDA, PLSVC-CS | Well |
|  | 65 | M | CoA, PLSVC-CS | SSRN, PFO, PLSVC-CS | Well |
|  | 68 | M | CoA | PFO | Well |
|  | 69 | F | CoA | SSRN | Well |
|  | 70 | M | CoA | ASD | Well |
|  | 71 | M | CoA | PFO | Well |
|  | 72 | M | CoA/HTAA | SSRN, PFO, ASD | ASD, PFO, Well |
|  | 73 | M | CoA,ASA/RFOF | SSRN | Well |
|  | 74 | M | CoA | PFO | Well |
|  | 75 | M | CoA, ASA/RFOF | ASD | ASD, Well |
|  | 76 | F | CoA | VSD, PFO | VSD, PFO, Well |
|  | 77 | M | CoA,ASA/RFOF | PFO, PDA | Well |
|  | 78 | M | CoA | SSRN, PFO, PDA | Well |
|  | 79 | F | CoA, PLSVC-CS, ASA/RFOF | SSRN, PFO, PLSVC-CS | PFO, PLSVC-CS, Well |
|  | 80 | M | CoA, VSD | PFO, PDA, VSD | PFO, PDA, Well |
|  | 81 | F | CoA, RFO | PFO | Well |
|  | 82 | M | CoA, PLSVC-CS | SSRN, PFO, PDA, PLSVC-CS | PLSVC-CS, Well |
| Confirmed CoA cases | 1 | M | CoA,BAV, mild AS | BAV, mild AS, PFO, PDA | Surgery, well |
|  | 3 | F | VSD,CoA or Aortic arch interuption, HTAA | IAA (type A),VSD,PDA,PFO | Surgery, well |
|  | 5 | M | CoA,VSD | CoA, VSD,PDA,PFO | Surgery, well |
|  | 6 | F | CoA/HTAA,PLSVC-CS | CoA, PLSVC-CS, PDA, PFO,PAH | Interventional therapy, well |
|  | 8 | F | CoA/HTAA,VSD | CoA, HTAA,VSD ASD | NND |
|  | 10 | F | CoA,VSD | CoA, VSD, PDA, ASD | Surgery, well |
|  | 16 | F | CoA,VSD | CoA,VSD,PDA,PFO,PAH | Surgery, well |
|  | 18 | F | CoA | CoA,PFO | Surgery, well |
|  | 26 | F | CoA,VSD | VSD, ASD, PAH | Surgery, well |
|  | 27 | F | CoA, | PFO, CoA | Surgery, well |
|  | 28 | M | CoA, ARSA | COA，PDA, PFO | Surgery, well |
|  | 31 | M | CoA | CoA, PFO | Surgery, well |
|  | 36 | M | CoA, PLSVC-CS | CoA,PDA,PFO, PLSVC-CS | Surgery, smaller left ventricle |
|  | 45 | F | CoA/HTAA | CoA/HTAA, PFO, ASD, PDA | Surgery, well, PFO |
|  | 46 | F | CoA, ASD | CoA, PFO, PDA, AVSD | Surgery, AS (4m/s), Ao arch 2.2m/s |
|  | 47 | M | CoA,VSD | CoA, PFO, PDA, VSD, PAH | Surgery, well |
|  | 48 | M | CoA,VSD | CoA, ASD, BAV, VSD | Surgery, BAV, Residual atrial shunt (1.5mm), well |
|  | 53 | M | CoA, PLSVC-CS | CoA, PDA, PFO, ASD, PLSVC-CS | Surgery, Ao arch 3.2m/s, PLSVC-CS |
|  | 54 | F | CoA, PLSVC-CS | CoA, PDA, PFO, ASD, PLSVC-CS | Surgery, well, PFO |
|  | 55 | F | CoA,VSD | CoA, ASD, VSD | Surgery, well |
|  | 58 | M | CoA | CoA,VSD | Surgery, well, PFO |
|  | 59 | F | CoA/HTAA, VSD | CoA, PDA, PFO, ASD, VSD | Interventional therapy, Ao arch 2.8m/s , VSD, PDA |
|  | 60 | M | CoA | CoA/HTAA, PFO, PDA | Surgery, well |
|  | 62 | F | CoA,VSD | CoA/HTAA, ASD, VSD, PFO, PDA | Surgery, Residual ventricular shunt (1.7mm), well |
|  | 63 | M | CoA/HTAA, PLSVC-CS, VSD | CoA/HTAA, VSD, PFO, PDA, PLSVC-CS | Surgery, Residual atrial shunt (3.0mm), well |
|  | 66 | F | CoA/HTAA, VSD, ARSA | CoA/HTAA, ASD, VSD, PDA, ARSA | Surgery, well |
|  | 67 | F | CoA, PLSVC-CS | CoA/HTAA, PDA, ASD, PLSVC-CS | Surgery, well |
|  | 83 | M | CoA, VSD | CoA, TAPVC, PFO, PDA, PAH | NND |

Abbreviations:

Ao, aorta; ARSA, aberrant right subclavian artery; AS, aortic valve stenosis; ASA, atrial septal aneurysm; ASD, atrial septal defect; AVSD, atrioventricular septal defect; BAV, bicuspid aortic valve; CoA, coarctation of the aorta; DA, ductus arteriosus; F, female; HTAA, hypoplastic transverse aortic arch; IAA, aortic arch interruption; LVIO, left ventricular inflow obstruction; M, male; NND, neonatal death; PAH, pulmonary arterial hypertension; PDA, patent ductus arteriosus; PFO, patent foramen ovale; PLSVC-CS, persistent left superior vena cava with coronary sinus dilation; PS, pulmonary valve stenosis; RFO, restrictive foramen ovale; RFOF, redundant foramen ovale flap; SSRN, some segments of aorta remains narrow; TAPVC, total anomalous pulmonary venous connection; VSD, ventricular septal defect.
